# Crystal structure of 1,4-dieth­oxy-9,10-anthra­quinone

**DOI:** 10.1107/S2056989015011901

**Published:** 2015-06-24

**Authors:** Chitoshi Kitamura, Sining Li, Munenori Takehara, Yoshinori Inoue, Katsuhiko Ono, Takeshi Kawase

**Affiliations:** a Department of Materials Science, School of Engineering, The University of Shiga Prefecture, 2500 Hassaka-cho, Hikone, Shiga 522-8533, Japan; b Department of Materials Science and Engineering, Graduate School of Engineering, Nagoya Institute of Technology, Gokiso, Showa-ku, Nagoya, Aichi 466-8555, Japan; c Department of Applied Chemistry, Graduate School of Engineering, University of Hyogo, 2167 Shosha, Himeji, Hyogo 671-2280, Japan

**Keywords:** crystal structure, 9,10-anthra­quinone, crystallographically independent mol­ecules, π–π inter­actions, C—H⋯O inter­actions

## Abstract

The asymmetric unit of the title compound, C_18_H_16_O_4_, contains two crystallographically independent mol­ecules. The anthra­quinone ring systems are slightly bent with dihedral angles of 2.33 (8) and 13.31 (9)° between the two terminal benzene rings. In the crystal, the two independent mol­ecules adopt slipped-parallel π-overlap with an average inter­planar distance of 3.45 Å, forming a dimer; the centroid–centroid distances of the π–π inter­actions are 3.6659 (15)–3.8987 (15) Å. The mol­ecules are also linked by C—H⋯O inter­actions, forming a tape structure along the *a-*axis direction. The crystal packing is characterized by a dimer-herringbone pattern.

## Related literature   

For synthesis of alk­oxy-substituted 9,10-anthra­quinones, see: Kitamura *et al.* (2004 ▸). For background information on substitution effects of alk­oxy-substituted 9,10-anthra­quinones, see; Ohta *et al.* (2012 ▸). For related structures of 1,4-diprop­oxy-9,10-anthra­quinone polymorphs, see: Kitamura *et al.* (2015 ▸).
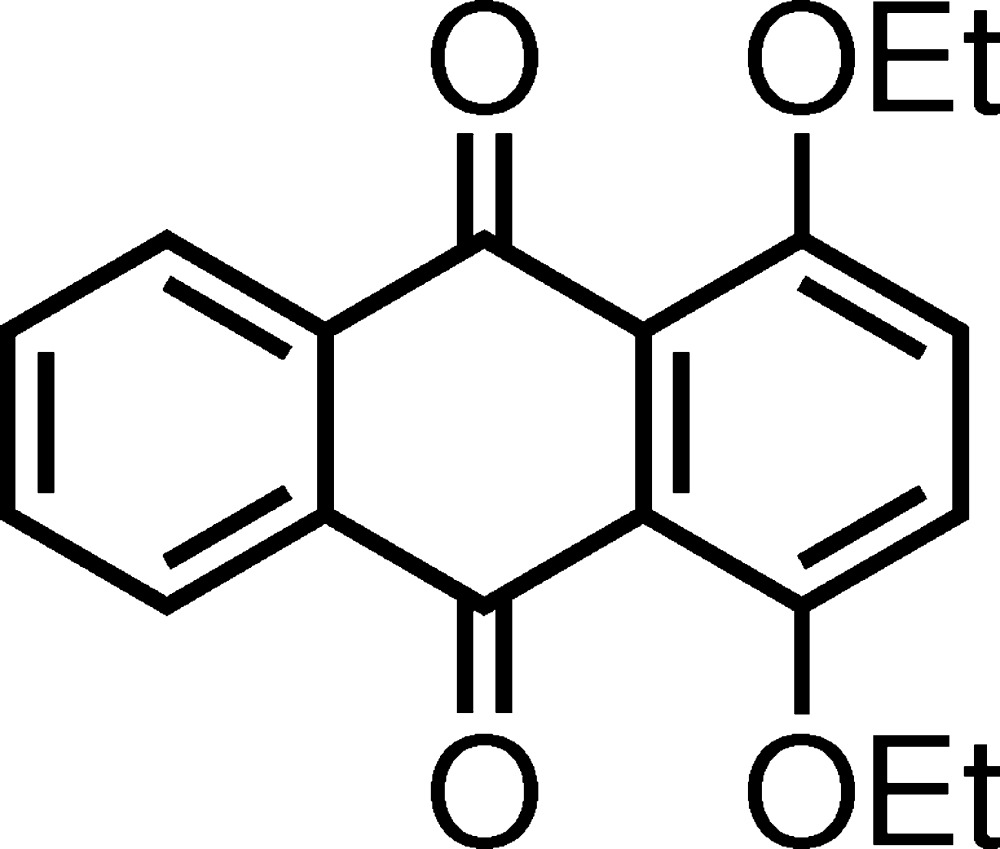


## Experimental   

### Crystal data  

C_18_H_16_O_4_
*M* *_r_* = 296.31
Monoclinic, 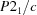

*a* = 13.5514 (11) Å
*b* = 14.7204 (11) Å
*c* = 14.5905 (10) Å
β = 90.604 (3)°
*V* = 2910.4 (4) Å^3^
*Z* = 8
Mo *K*α radiation
μ = 0.10 mm^−1^
*T* = 223 K
0.56 × 0.40 × 0.36 mm

### Data collection  

Rigaku R-AXIS RAPID diffractometer
27699 measured reflections
6645 independent reflections
3129 reflections with *I* > 2σ(*I*)
*R*_int_ = 0.045

### Refinement  

*R*[*F* ^2^ > 2σ(*F* ^2^)] = 0.076
*wR*(*F* ^2^) = 0.273
*S* = 0.93
6645 reflections
397 parameters
H-atom parameters constrained
Δρ_max_ = 0.27 e Å^−3^
Δρ_min_ = −0.48 e Å^−3^

### 

Data collection: *PROCESS-AUTO* (Rigaku, 1998 ▸); cell refinement: *PROCESS-AUTO*; data reduction: *PROCESS-AUTO*; program(s) used to solve structure: *SIR2004* (Burla *et al.*, 2005 ▸); program(s) used to refine structure: *SHELXL2014* (Sheldrick, 2015 ▸); molecular graphics: *ORTEP-3 for Windows* (Farrugia, 2012 ▸) and *Mercury* (Macrae *et al.*, 2008 ▸); software used to prepare material for publication: *WinGX* (Farrugia, 2012 ▸).

## Supplementary Material

Crystal structure: contains datablock(s) I, global. DOI: 10.1107/S2056989015011901/is5404sup1.cif

Structure factors: contains datablock(s) I. DOI: 10.1107/S2056989015011901/is5404Isup2.hkl

Supporting information file. DOI: 10.1107/S2056989015011901/is5404Isup3.cml

**Fig. 1.**
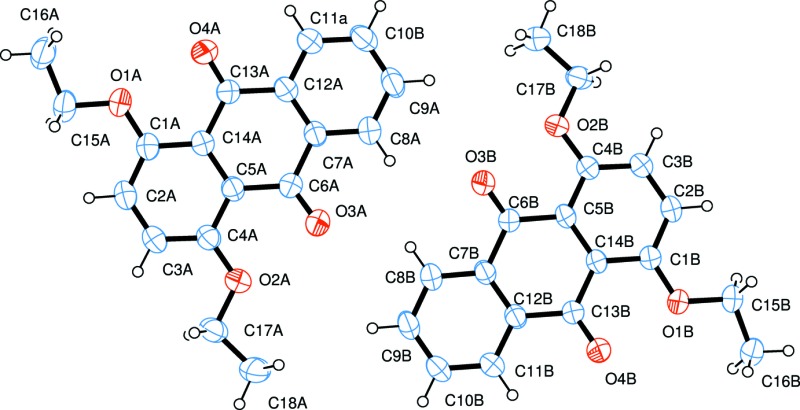
The asymmetric unit of the title compound, showing the atomic numbering and 40% probability displacement ellipsoids.

**Fig. 2.**
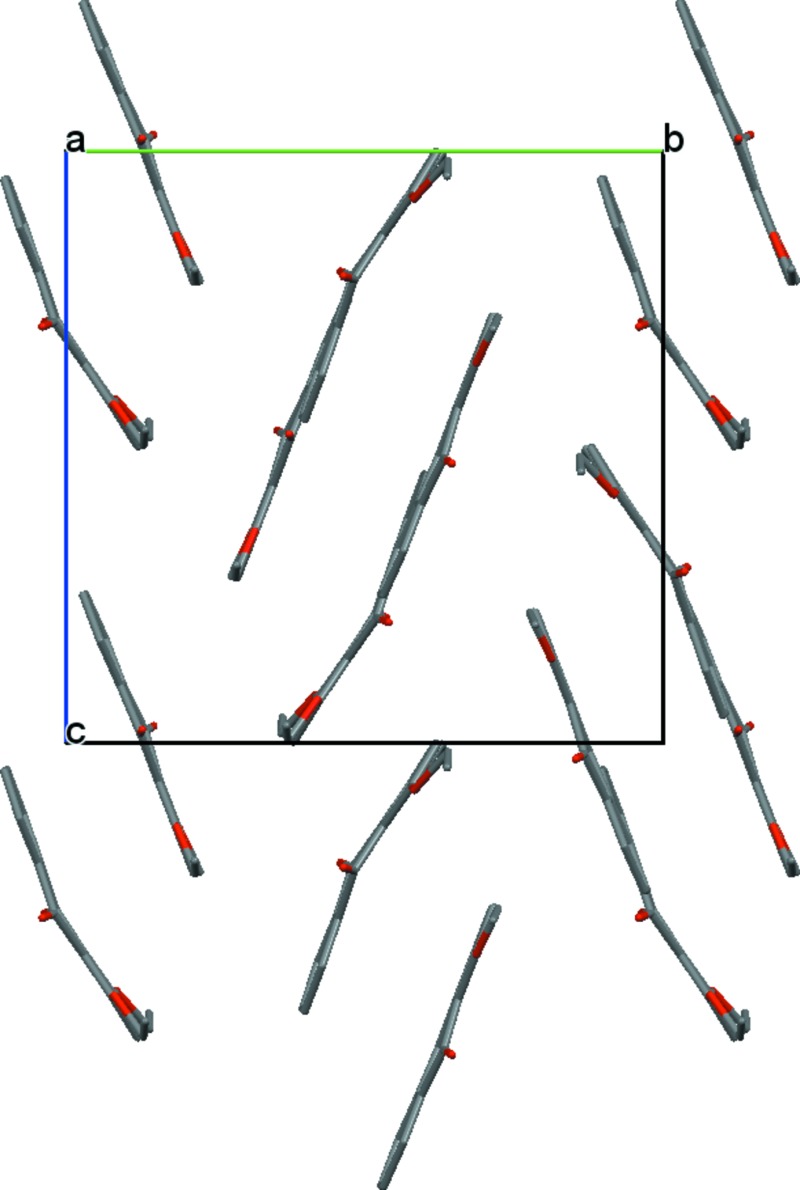
A packing diagram of the title compound viewed down the a axis, showing a dimer-herringbone pattern. Hydrogen atoms are omitted for clarity.

. DOI: 10.1107/S2056989015011901/is5404fig1.tif
The asymmetric unit of the title compound, showing the atomic numbering and 40% probability displacement ellipsoids.

a . DOI: 10.1107/S2056989015011901/is5404fig2.tif
A packing diagram of the title compound viewed down the *a* axis, showing a dimer-herringbone pattern. Hydrogen atoms are omitted for clarity.

**Fig. 3.**
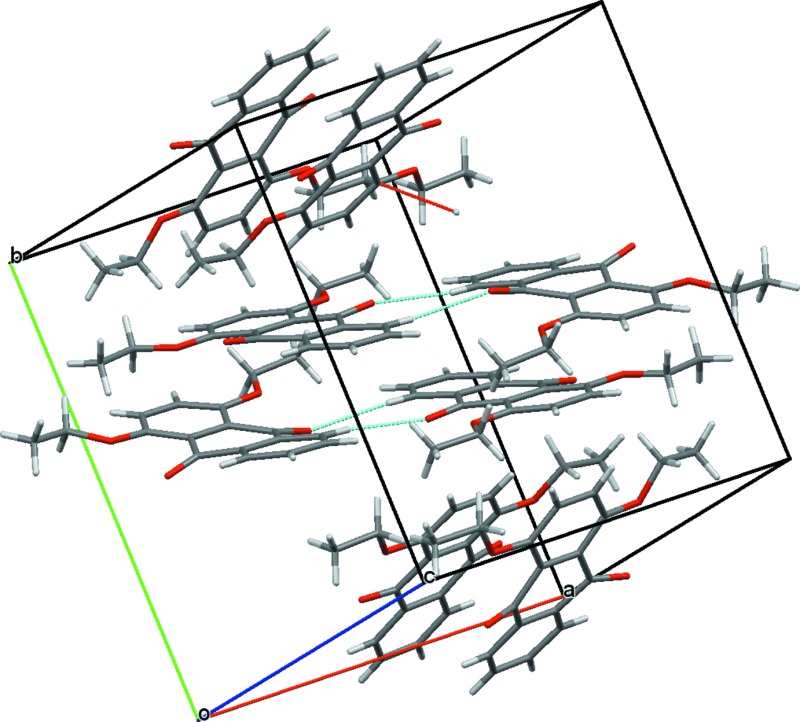
A packing diagram of the title compound, showing C—H···O interactions (blue lines).

. DOI: 10.1107/S2056989015011901/is5404fig3.tif
A packing diagram of the title compound, showing C—H⋯O inter­actions (blue lines).

CCDC reference: 1008606

Additional supporting information:  crystallographic information; 3D view; checkCIF report

## Figures and Tables

**Table 1:** Hydrogen-bond geometry (, )

*D*H*A*	*D*H	H*A*	*D* *A*	*D*H*A*
C8*A*H8*A*O3*B*	0.94	2.48	3.234(3)	137
C8*B*H8*B*O3*A*	0.94	2.55	3.304(4)	137
C11*A*H11*A*O4*B* ^i^	0.94	2.60	3.325(3)	135
C11*B*H11*B*O4*A* ^ii^	0.94	2.46	3.199(4)	135

Symmetry codes: (i) 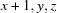; (ii) 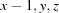.

## supplementary crystallographic information

### S1. Comment

9,10-Anthraquinone is an important framework as a dye. Various kinds of hydroxy-substituted anthraquinone dyes have been manufactured. However, there were little reports on alkoxy-substituted anthraquinone. In recent years, we presented the effects of the alkoxy substitution on the optical properties of 2,6-dialkoxy and 2,3,6,7-tetraalkoxy derivatives in solution as well as in the solid state (Ohta *et al.*, 2012). Very recently, we have reported crystal structures of two polymorphs of 1,4-dipropoxy-9,10-anthraquinone, which contained red and yellow solids (Kitamura *et al.*, 2015). The red crystal exhibited an anti-parallel arrangement along the stacking direction. On the other hand, the yellow crystal showed a slipped-parallel arrangement. To search the effect of alkyl chain length on molecular packing, we prepared the title compound, 1,4-diethoxy-9,10-anthraquinone, (I). In this paper, we present the crystal structure of (I).

The molecular structure of (I) is shown in Fig. 1. Two crystallographically independent molecules were found in the asymmetric unit, although the two molecules had almost the same molecular structure. There was a difference in planarity between the two molecules. Thus, the anthraquinone framework was slightly bent at the central quinone ring. For example, the dihedral angle between the two terminal benzene rings in the anthraquinone was 2.33 (8)° for one molecule and 13.31 (9)° for the other. The packing structure displays a dimer-herringbone pattern (Fig. 2), which is completely different from those of 1,4-dipropoxy-9,10-anthraquinone polymorphs (Kitamura *et al.*, 2015). In the dimer part, the two molecules adopt slipped-parallel π-stack with an average interplanar distance of 3.45 Å, which would result in a yellow color in the solid state. The crystal structure is also stabilized by C—H···O interactions along the lateral direction of molecules (Fig. 3).

### S2. Experimental

The title compound was prepared according to our previously reported method (Kitamura *et al.*, 2004). A mixture of 1,4-hydrooxy-9,10-anthraquinone (2.20 g, 9.16 mmol), K_2_CO_3_ (2.51 g, 18.1 mmol), ethyl *p*-toluenesulfonate (5.02 g, 25.1 mmol) in *o*-dichlorobenzene (15 ml) was heated at reflux for 3 h under N_2_ gas. After cooling to room temperature, water (65 ml) was added to the reaction mixture. Then, the resulting solid was filtered off and washed with hexane to give the title compound (2.37 g, 87% yield) as a yellow solid. Single crystals suitable for X-ray analysis were obtained by slow evaporation from a CH_2_Cl_2_ solution (*m*.*p*. 172–175 °C). Elemental analysis for C_18_H_16_O_4_: C 72.96, H 5.44. Found: C 72.75, H 5.51. TOF-MS(EI): *m*/*z* Calcd C_18_H_16_O_4_: 296.1049. Found: 296.1074.

### S3. Refinement

All the H atoms were positioned geometrically and refined using a riding model with C—H bonds of 0.94 Å, 0.98 Å, and 0.97 Å for aromatic, methylene and methyl groups, respectively, and *U*_iso_(H) = 1.2*U*_eq_(C) [*U*_iso_(H) = 1.5*U*_eq_(C) for methyl H atoms].

### Crystal data

**Table:**

C_18_H_16_O_4_	*F*(000) = 1248
*M**_r_* = 296.31	*D*_x_ = 1.352 Mg m^−^^3^
Monoclinic, *P*2_1_/*c*	Mo *K*α radiation, λ = 0.71073 Å
Hall symbol: -P 2ybc	Cell parameters from 11158 reflections
*a* = 13.5514 (11) Å	θ = 3–27.5°
*b* = 14.7204 (11) Å	µ = 0.10 mm^−^^1^
*c* = 14.5905 (10) Å	*T* = 223 K
β = 90.604 (3)°	Prism, orange
*V* = 2910.4 (4) Å^3^	0.56 × 0.40 × 0.36 mm
*Z* = 8	

### Data collection

**Table:**

Rigaku R-AXIS RAPID diffractometer	3129 reflections with *I* > 2σ(*I*)
Radiation source: fine-focus sealed x-ray tube	*R*_int_ = 0.045
Graphite monochromator	θ_max_ = 27.5°, θ_min_ = 3.0°
Detector resolution: 10 pixels mm^-1^	*h* = −17→17
ω scans	*k* = −19→19
27699 measured reflections	*l* = −16→18
6645 independent reflections	

### Refinement

**Table:**

Refinement on *F*^2^	0 restraints
Least-squares matrix: full	0 constraints
*R*[*F*^2^ > 2σ(*F*^2^)] = 0.076	H-atom parameters constrained
*wR*(*F*^2^) = 0.273	*w* = 1/[σ^2^(*F*_o_^2^) + (0.1824*P*)^2^] where *P* = (*F*_o_^2^ + 2*F*_c_^2^)/3
*S* = 0.93	(Δ/σ)_max_ < 0.001
6645 reflections	Δρ_max_ = 0.27 e Å^−^^3^
397 parameters	Δρ_min_ = −0.48 e Å^−^^3^

### Special details

**Table:**

Experimental. ^1^H-NMR: *δ* 1.56 (t, *J* = 7.0 Hz, 6H), 4.20 (q, *J* = 7.0 Hz, 4H), 7.32 (s, 2H), 7.69–7.72 (m, 2H), 8.17–8.19 (m, 2H); ^13^C-NMR: *δ* 14.9, 66.0, 122.1, 123.4, 126.4, 133.2, 134.2, 153.6, 183.3.
Geometry. All e.s.d.'s (except the e.s.d. in the dihedral angle between two l.s. planes) are estimated using the full covariance matrix. The cell e.s.d.'s are taken into account individually in the estimation of e.s.d.'s in distances, angles and torsion angles; correlations between e.s.d.'s in cell parameters are only used when they are defined by crystal symmetry. An approximate (isotropic) treatment of cell e.s.d.'s is used for estimating e.s.d.'s involving l.s. planes.

### Fractional atomic coordinates and isotropic or equivalent isotropic displacement parameters (Å^2^)

**Table:**

	*x*	*y*	*z*	*U*_iso_*/*U*_eq_	
C1A	0.3813 (2)	0.68438 (17)	0.35528 (16)	0.0548 (6)	
C2A	0.3271 (2)	0.71337 (18)	0.27977 (17)	0.0611 (7)	
H2A	0.3605	0.7327	0.2271	0.073*	
C3A	0.2261 (2)	0.71464 (18)	0.27990 (16)	0.0596 (7)	
H3A	0.1918	0.7347	0.2274	0.072*	
C4A	0.1731 (2)	0.68680 (17)	0.35617 (16)	0.0546 (6)	
C5A	0.22559 (19)	0.65664 (16)	0.43427 (16)	0.0518 (6)	
C6A	0.1713 (2)	0.63214 (19)	0.51927 (18)	0.0611 (7)	
C7A	0.22803 (19)	0.59444 (17)	0.59796 (15)	0.0527 (6)	
C8A	0.1770 (2)	0.56507 (18)	0.67540 (17)	0.0632 (7)	
H8A	0.1078	0.5678	0.6766	0.076*	
C9A	0.2293 (2)	0.5320 (2)	0.75003 (17)	0.0707 (8)	
H9A	0.1952	0.5121	0.8021	0.085*	
C10A	0.3301 (2)	0.5279 (2)	0.74903 (18)	0.0726 (8)	
H10A	0.3647	0.5057	0.8005	0.087*	
C11A	0.3814 (2)	0.5564 (2)	0.67253 (18)	0.0681 (8)	
H11A	0.4507	0.553	0.6717	0.082*	
C12A	0.3296 (2)	0.59013 (18)	0.59665 (17)	0.0572 (6)	
C13A	0.3855 (2)	0.6237 (2)	0.5163 (2)	0.0734 (9)	
C14A	0.32994 (19)	0.65421 (16)	0.43355 (16)	0.0530 (6)	
C15A	0.5325 (2)	0.7171 (2)	0.2792 (2)	0.0782 (9)	
H15A	0.5119	0.7793	0.265	0.094*	
H15B	0.5174	0.6787	0.226	0.094*	
C16A	0.6399 (2)	0.7146 (2)	0.2998 (2)	0.0814 (9)	
H16A	0.676	0.7365	0.2471	0.122*	
H16B	0.6542	0.7531	0.3523	0.122*	
H16C	0.6598	0.6527	0.3134	0.122*	
C17A	0.0200 (2)	0.7234 (2)	0.28108 (18)	0.0672 (7)	
H17A	0.0363	0.6897	0.2253	0.081*	
H17B	0.0373	0.7874	0.2719	0.081*	
C18A	−0.0867 (2)	0.7146 (2)	0.3010 (2)	0.0787 (9)	
H18A	−0.1251	0.7386	0.25	0.118*	
H18B	−0.1029	0.651	0.3099	0.118*	
H18C	−0.1019	0.7483	0.3562	0.118*	
O1A	0.48108 (14)	0.68464 (14)	0.35759 (12)	0.0673 (5)	
O2A	0.07320 (14)	0.68735 (14)	0.35765 (12)	0.0670 (5)	
O3A	0.08375 (17)	0.6462 (2)	0.52801 (15)	0.1020 (9)	
O4A	0.47401 (18)	0.6270 (3)	0.5216 (2)	0.1549 (16)	
C1B	−0.31908 (18)	0.42295 (17)	0.93060 (15)	0.0515 (6)	
C2B	−0.2634 (2)	0.3807 (2)	0.99876 (16)	0.0600 (7)	
H2B	−0.296	0.3526	1.0478	0.072*	
C3B	−0.1627 (2)	0.37894 (19)	0.99648 (16)	0.0582 (7)	
H3B	−0.1276	0.3486	1.0432	0.07*	
C4B	−0.11091 (18)	0.42118 (16)	0.92621 (15)	0.0507 (6)	
C5B	−0.16423 (18)	0.46877 (16)	0.85830 (14)	0.0477 (6)	
C6B	−0.11215 (18)	0.51742 (17)	0.78342 (15)	0.0517 (6)	
C7B	−0.17014 (18)	0.54479 (16)	0.70136 (15)	0.0495 (6)	
C8B	−0.1216 (2)	0.57178 (19)	0.62221 (17)	0.0636 (7)	
H8B	−0.0523	0.5707	0.6203	0.076*	
C9B	−0.1754 (2)	0.6001 (2)	0.54668 (17)	0.0683 (8)	
H9B	−0.1426	0.6169	0.4928	0.082*	
C10B	−0.2760 (2)	0.6040 (2)	0.54962 (17)	0.0680 (8)	
H10B	−0.312	0.6241	0.498	0.082*	
C11B	−0.3252 (2)	0.57867 (18)	0.62810 (17)	0.0634 (7)	
H11B	−0.3944	0.5823	0.63	0.076*	
C12B	−0.27238 (18)	0.54778 (16)	0.70427 (15)	0.0499 (6)	
C13B	−0.32523 (18)	0.52046 (17)	0.78847 (15)	0.0520 (6)	
C14B	−0.26902 (17)	0.47017 (16)	0.86019 (14)	0.0474 (5)	
C15B	−0.4706 (2)	0.3711 (2)	0.99707 (18)	0.0672 (8)	
H15C	−0.462	0.4013	1.0565	0.081*	
H15D	−0.4444	0.3092	1.0022	0.081*	
C16B	−0.5768 (2)	0.3682 (2)	0.97143 (19)	0.0714 (8)	
H16D	−0.6129	0.3351	1.0178	0.107*	
H16E	−0.6022	0.4296	0.9669	0.107*	
H16F	−0.5847	0.3378	0.9128	0.107*	
C17B	0.04137 (19)	0.36059 (19)	0.98395 (17)	0.0605 (7)	
H17C	0.0159	0.2983	0.9816	0.073*	
H17D	0.0335	0.3839	1.0464	0.073*	
C18B	0.1478 (2)	0.3621 (2)	0.95790 (19)	0.0681 (7)	
H18D	0.1855	0.3244	1.0001	0.102*	
H18E	0.1546	0.3388	0.8961	0.102*	
H18F	0.1722	0.424	0.9606	0.102*	
O1B	−0.41883 (13)	0.42021 (13)	0.92745 (11)	0.0609 (5)	
O2B	−0.01133 (12)	0.41697 (12)	0.91984 (11)	0.0586 (5)	
O3B	−0.02445 (14)	0.53591 (16)	0.78861 (12)	0.0763 (6)	
O4B	−0.41212 (14)	0.54036 (16)	0.79662 (13)	0.0792 (6)	

### Atomic displacement parameters (Å^2^)

**Table:**

	*U*^11^	*U*^22^	*U*^33^	*U*^12^	*U*^13^	*U*^23^
C1A	0.0605 (17)	0.0546 (14)	0.0495 (13)	−0.0036 (11)	0.0078 (11)	−0.0019 (11)
C2A	0.0725 (19)	0.0649 (16)	0.0460 (13)	−0.0059 (13)	0.0096 (12)	0.0024 (11)
C3A	0.0730 (19)	0.0623 (15)	0.0436 (13)	−0.0014 (13)	−0.0008 (12)	0.0022 (11)
C4A	0.0588 (17)	0.0563 (14)	0.0488 (13)	−0.0021 (11)	−0.0015 (11)	0.0009 (11)
C5A	0.0555 (15)	0.0521 (13)	0.0478 (13)	0.0009 (10)	0.0027 (11)	0.0039 (10)
C6A	0.0500 (16)	0.0738 (18)	0.0595 (15)	0.0006 (12)	0.0044 (12)	0.0125 (13)
C7A	0.0571 (16)	0.0554 (14)	0.0456 (13)	−0.0020 (11)	0.0035 (11)	0.0040 (10)
C8A	0.0642 (18)	0.0737 (17)	0.0519 (14)	−0.0018 (13)	0.0090 (12)	0.0057 (12)
C9A	0.085 (2)	0.0814 (19)	0.0452 (14)	−0.0047 (16)	0.0060 (13)	0.0117 (13)
C10A	0.080 (2)	0.087 (2)	0.0507 (15)	−0.0024 (16)	−0.0112 (13)	0.0135 (14)
C11A	0.0623 (18)	0.0812 (19)	0.0605 (16)	−0.0011 (13)	−0.0049 (13)	0.0164 (14)
C12A	0.0600 (17)	0.0620 (15)	0.0497 (13)	−0.0015 (12)	−0.0007 (11)	0.0084 (11)
C13A	0.0499 (17)	0.102 (2)	0.0680 (17)	−0.0035 (15)	0.0006 (13)	0.0333 (16)
C14A	0.0550 (15)	0.0549 (14)	0.0490 (13)	−0.0013 (11)	0.0026 (11)	0.0048 (10)
C15A	0.072 (2)	0.100 (2)	0.0625 (17)	−0.0060 (17)	0.0221 (15)	0.0049 (16)
C16A	0.068 (2)	0.099 (2)	0.078 (2)	−0.0059 (17)	0.0214 (16)	0.0005 (17)
C17A	0.0692 (19)	0.0800 (19)	0.0522 (14)	0.0096 (14)	−0.0100 (12)	0.0001 (13)
C18A	0.068 (2)	0.102 (2)	0.0660 (17)	0.0023 (16)	−0.0154 (14)	−0.0043 (16)
O1A	0.0570 (12)	0.0860 (13)	0.0593 (11)	−0.0029 (9)	0.0144 (9)	0.0075 (9)
O2A	0.0574 (12)	0.0872 (13)	0.0563 (10)	−0.0004 (9)	−0.0067 (8)	0.0116 (9)
O3A	0.0555 (14)	0.165 (3)	0.0855 (15)	0.0170 (14)	0.0145 (11)	0.0563 (15)
O4A	0.0496 (16)	0.289 (4)	0.126 (2)	−0.0097 (19)	−0.0050 (14)	0.127 (3)
C1B	0.0491 (14)	0.0616 (14)	0.0440 (12)	−0.0026 (11)	0.0030 (10)	0.0047 (10)
C2B	0.0609 (17)	0.0720 (17)	0.0473 (13)	−0.0048 (13)	0.0066 (12)	0.0168 (12)
C3B	0.0557 (16)	0.0688 (16)	0.0500 (13)	0.0000 (12)	−0.0007 (11)	0.0154 (12)
C4B	0.0514 (15)	0.0590 (14)	0.0416 (12)	0.0001 (11)	−0.0002 (10)	0.0020 (10)
C5B	0.0534 (14)	0.0556 (13)	0.0342 (11)	−0.0005 (10)	0.0029 (9)	0.0021 (9)
C6B	0.0455 (14)	0.0653 (15)	0.0442 (12)	−0.0025 (11)	0.0026 (10)	0.0057 (11)
C7B	0.0543 (15)	0.0537 (13)	0.0407 (11)	−0.0022 (10)	0.0020 (10)	0.0059 (10)
C8B	0.0624 (17)	0.0802 (18)	0.0483 (13)	−0.0060 (14)	0.0066 (12)	0.0134 (12)
C9B	0.075 (2)	0.085 (2)	0.0451 (13)	−0.0091 (15)	0.0059 (13)	0.0173 (13)
C10B	0.074 (2)	0.0826 (19)	0.0474 (14)	−0.0010 (15)	−0.0059 (13)	0.0200 (13)
C11B	0.0597 (17)	0.0770 (18)	0.0534 (14)	0.0034 (13)	−0.0030 (12)	0.0164 (13)
C12B	0.0546 (15)	0.0539 (13)	0.0413 (12)	0.0013 (10)	0.0028 (10)	0.0056 (10)
C13B	0.0475 (14)	0.0630 (15)	0.0455 (12)	0.0023 (11)	0.0027 (10)	0.0063 (11)
C14B	0.0511 (14)	0.0551 (13)	0.0359 (11)	0.0002 (10)	0.0036 (9)	0.0007 (9)
C15B	0.0576 (18)	0.0847 (19)	0.0596 (16)	−0.0086 (14)	0.0113 (13)	0.0203 (14)
C16B	0.0570 (18)	0.095 (2)	0.0620 (16)	−0.0103 (15)	0.0097 (13)	0.0086 (15)
C17B	0.0567 (17)	0.0672 (16)	0.0572 (14)	−0.0002 (12)	−0.0105 (12)	0.0086 (12)
C18B	0.0550 (17)	0.0804 (19)	0.0688 (17)	0.0069 (14)	−0.0070 (13)	0.0045 (14)
O1B	0.0471 (11)	0.0822 (13)	0.0535 (10)	−0.0018 (8)	0.0076 (8)	0.0159 (8)
O2B	0.0478 (11)	0.0773 (12)	0.0508 (9)	0.0029 (8)	−0.0036 (7)	0.0139 (8)
O3B	0.0512 (12)	0.1171 (17)	0.0605 (11)	−0.0142 (11)	−0.0020 (8)	0.0288 (11)
O4B	0.0525 (12)	0.1193 (17)	0.0659 (12)	0.0183 (11)	0.0086 (9)	0.0321 (11)

### Geometric parameters (Å, º)

**Table:**

C1A—O1A	1.352 (3)	C1B—O1B	1.353 (3)
C1A—C2A	1.385 (4)	C1B—C2B	1.388 (3)
C1A—C14A	1.415 (3)	C1B—C14B	1.419 (3)
C2A—C3A	1.368 (4)	C2B—C3B	1.366 (3)
C2A—H2A	0.94	C2B—H2B	0.94
C3A—C4A	1.392 (3)	C3B—C4B	1.395 (3)
C3A—H3A	0.94	C3B—H3B	0.94
C4A—O2A	1.354 (3)	C4B—O2B	1.355 (3)
C4A—C5A	1.409 (3)	C4B—C5B	1.407 (3)
C5A—C14A	1.415 (3)	C5B—C14B	1.421 (3)
C5A—C6A	1.493 (3)	C5B—C6B	1.490 (3)
C6A—O3A	1.212 (3)	C6B—O3B	1.221 (3)
C6A—C7A	1.483 (3)	C6B—C7B	1.481 (3)
C7A—C12A	1.378 (4)	C7B—C12B	1.387 (3)
C7A—C8A	1.399 (3)	C7B—C8B	1.393 (3)
C8A—C9A	1.381 (4)	C8B—C9B	1.379 (4)
C8A—H8A	0.94	C8B—H8B	0.94
C9A—C10A	1.367 (4)	C9B—C10B	1.366 (4)
C9A—H9A	0.94	C9B—H9B	0.94
C10A—C11A	1.386 (4)	C10B—C11B	1.383 (4)
C10A—H10A	0.94	C10B—H10B	0.94
C11A—C12A	1.396 (4)	C11B—C12B	1.392 (3)
C11A—H11A	0.94	C11B—H11B	0.94
C12A—C13A	1.488 (4)	C12B—C13B	1.484 (3)
C13A—O4A	1.202 (3)	C13B—O4B	1.220 (3)
C13A—C14A	1.485 (4)	C13B—C14B	1.485 (3)
C15A—O1A	1.428 (3)	C15B—O1B	1.436 (3)
C15A—C16A	1.484 (4)	C15B—C16B	1.484 (4)
C15A—H15A	0.98	C15B—H15C	0.98
C15A—H15B	0.98	C15B—H15D	0.98
C16A—H16A	0.97	C16B—H16D	0.97
C16A—H16B	0.97	C16B—H16E	0.97
C16A—H16C	0.97	C16B—H16F	0.97
C17A—O2A	1.425 (3)	C17B—O2B	1.435 (3)
C17A—C18A	1.485 (4)	C17B—C18B	1.495 (4)
C17A—H17A	0.98	C17B—H17C	0.98
C17A—H17B	0.98	C17B—H17D	0.98
C18A—H18A	0.97	C18B—H18D	0.97
C18A—H18B	0.97	C18B—H18E	0.97
C18A—H18C	0.97	C18B—H18F	0.97
			
O1A—C1A—C2A	122.8 (2)	O1B—C1B—C2B	123.1 (2)
O1A—C1A—C14A	118.8 (2)	O1B—C1B—C14B	118.4 (2)
C2A—C1A—C14A	118.5 (3)	C2B—C1B—C14B	118.5 (2)
C3A—C2A—C1A	121.7 (2)	C3B—C2B—C1B	121.8 (2)
C3A—C2A—H2A	119.2	C3B—C2B—H2B	119.1
C1A—C2A—H2A	119.2	C1B—C2B—H2B	119.1
C2A—C3A—C4A	121.4 (2)	C2B—C3B—C4B	121.4 (2)
C2A—C3A—H3A	119.3	C2B—C3B—H3B	119.3
C4A—C3A—H3A	119.3	C4B—C3B—H3B	119.3
O2A—C4A—C3A	122.3 (2)	O2B—C4B—C3B	122.6 (2)
O2A—C4A—C5A	119.0 (2)	O2B—C4B—C5B	118.6 (2)
C3A—C4A—C5A	118.6 (3)	C3B—C4B—C5B	118.7 (2)
C4A—C5A—C14A	119.9 (2)	C4B—C5B—C14B	120.0 (2)
C4A—C5A—C6A	119.9 (2)	C4B—C5B—C6B	120.8 (2)
C14A—C5A—C6A	120.1 (2)	C14B—C5B—C6B	119.24 (19)
O3A—C6A—C7A	118.9 (2)	O3B—C6B—C7B	119.8 (2)
O3A—C6A—C5A	122.5 (2)	O3B—C6B—C5B	122.0 (2)
C7A—C6A—C5A	118.5 (2)	C7B—C6B—C5B	118.2 (2)
C12A—C7A—C8A	119.9 (2)	C12B—C7B—C8B	119.8 (2)
C12A—C7A—C6A	121.1 (2)	C12B—C7B—C6B	120.4 (2)
C8A—C7A—C6A	119.0 (2)	C8B—C7B—C6B	119.8 (2)
C9A—C8A—C7A	119.5 (3)	C9B—C8B—C7B	119.9 (3)
C9A—C8A—H8A	120.3	C9B—C8B—H8B	120
C7A—C8A—H8A	120.3	C7B—C8B—H8B	120
C10A—C9A—C8A	120.7 (3)	C10B—C9B—C8B	120.4 (2)
C10A—C9A—H9A	119.6	C10B—C9B—H9B	119.8
C8A—C9A—H9A	119.6	C8B—C9B—H9B	119.8
C9A—C10A—C11A	120.3 (3)	C9B—C10B—C11B	120.3 (2)
C9A—C10A—H10A	119.8	C9B—C10B—H10B	119.8
C11A—C10A—H10A	119.8	C11B—C10B—H10B	119.8
C10A—C11A—C12A	119.6 (3)	C10B—C11B—C12B	120.0 (3)
C10A—C11A—H11A	120.2	C10B—C11B—H11B	120
C12A—C11A—H11A	120.2	C12B—C11B—H11B	120
C7A—C12A—C11A	120.0 (2)	C7B—C12B—C11B	119.4 (2)
C7A—C12A—C13A	120.8 (2)	C7B—C12B—C13B	120.5 (2)
C11A—C12A—C13A	119.2 (3)	C11B—C12B—C13B	120.0 (2)
O4A—C13A—C14A	122.5 (3)	O4B—C13B—C12B	119.3 (2)
O4A—C13A—C12A	118.6 (3)	O4B—C13B—C14B	122.6 (2)
C14A—C13A—C12A	118.9 (2)	C12B—C13B—C14B	118.0 (2)
C5A—C14A—C1A	119.9 (2)	C1B—C14B—C5B	119.5 (2)
C5A—C14A—C13A	120.0 (2)	C1B—C14B—C13B	120.6 (2)
C1A—C14A—C13A	120.0 (2)	C5B—C14B—C13B	119.9 (2)
O1A—C15A—C16A	108.4 (3)	O1B—C15B—C16B	108.4 (2)
O1A—C15A—H15A	110	O1B—C15B—H15C	110
C16A—C15A—H15A	110	C16B—C15B—H15C	110
O1A—C15A—H15B	110	O1B—C15B—H15D	110
C16A—C15A—H15B	110	C16B—C15B—H15D	110
H15A—C15A—H15B	108.4	H15C—C15B—H15D	108.4
C15A—C16A—H16A	109.5	C15B—C16B—H16D	109.5
C15A—C16A—H16B	109.5	C15B—C16B—H16E	109.5
H16A—C16A—H16B	109.5	H16D—C16B—H16E	109.5
C15A—C16A—H16C	109.5	C15B—C16B—H16F	109.5
H16A—C16A—H16C	109.5	H16D—C16B—H16F	109.5
H16B—C16A—H16C	109.5	H16E—C16B—H16F	109.5
O2A—C17A—C18A	107.4 (2)	O2B—C17B—C18B	107.5 (2)
O2A—C17A—H17A	110.2	O2B—C17B—H17C	110.2
C18A—C17A—H17A	110.2	C18B—C17B—H17C	110.2
O2A—C17A—H17B	110.2	O2B—C17B—H17D	110.2
C18A—C17A—H17B	110.2	C18B—C17B—H17D	110.2
H17A—C17A—H17B	108.5	H17C—C17B—H17D	108.5
C17A—C18A—H18A	109.5	C17B—C18B—H18D	109.5
C17A—C18A—H18B	109.5	C17B—C18B—H18E	109.5
H18A—C18A—H18B	109.5	H18D—C18B—H18E	109.5
C17A—C18A—H18C	109.5	C17B—C18B—H18F	109.5
H18A—C18A—H18C	109.5	H18D—C18B—H18F	109.5
H18B—C18A—H18C	109.5	H18E—C18B—H18F	109.5
C1A—O1A—C15A	118.5 (2)	C1B—O1B—C15B	119.11 (19)
C4A—O2A—C17A	119.1 (2)	C4B—O2B—C17B	118.09 (19)
			
O1A—C1A—C2A—C3A	−178.3 (2)	O1B—C1B—C2B—C3B	175.1 (2)
C14A—C1A—C2A—C3A	1.0 (4)	C14B—C1B—C2B—C3B	−4.0 (4)
C1A—C2A—C3A—C4A	0.0 (4)	C1B—C2B—C3B—C4B	1.3 (4)
C2A—C3A—C4A—O2A	−179.9 (2)	C2B—C3B—C4B—O2B	−176.9 (2)
C2A—C3A—C4A—C5A	−0.2 (4)	C2B—C3B—C4B—C5B	2.0 (4)
O2A—C4A—C5A—C14A	179.0 (2)	O2B—C4B—C5B—C14B	176.5 (2)
C3A—C4A—C5A—C14A	−0.7 (4)	C3B—C4B—C5B—C14B	−2.4 (3)
O2A—C4A—C5A—C6A	−4.3 (4)	O2B—C4B—C5B—C6B	−3.0 (3)
C3A—C4A—C5A—C6A	176.0 (2)	C3B—C4B—C5B—C6B	178.1 (2)
C4A—C5A—C6A—O3A	−8.2 (4)	C4B—C5B—C6B—O3B	−17.1 (4)
C14A—C5A—C6A—O3A	168.5 (3)	C14B—C5B—C6B—O3B	163.4 (2)
C4A—C5A—C6A—C7A	175.7 (2)	C4B—C5B—C6B—C7B	163.8 (2)
C14A—C5A—C6A—C7A	−7.6 (4)	C14B—C5B—C6B—C7B	−15.7 (3)
O3A—C6A—C7A—C12A	−170.0 (3)	O3B—C6B—C7B—C12B	−161.9 (2)
C5A—C6A—C7A—C12A	6.3 (4)	C5B—C6B—C7B—C12B	17.2 (3)
O3A—C6A—C7A—C8A	8.4 (4)	O3B—C6B—C7B—C8B	15.1 (4)
C5A—C6A—C7A—C8A	−175.4 (2)	C5B—C6B—C7B—C8B	−165.8 (2)
C12A—C7A—C8A—C9A	0.1 (4)	C12B—C7B—C8B—C9B	−0.9 (4)
C6A—C7A—C8A—C9A	−178.3 (2)	C6B—C7B—C8B—C9B	−177.9 (2)
C7A—C8A—C9A—C10A	0.1 (4)	C7B—C8B—C9B—C10B	1.6 (4)
C8A—C9A—C10A—C11A	−0.5 (5)	C8B—C9B—C10B—C11B	−0.7 (5)
C9A—C10A—C11A—C12A	0.6 (5)	C9B—C10B—C11B—C12B	−0.9 (4)
C8A—C7A—C12A—C11A	0.1 (4)	C8B—C7B—C12B—C11B	−0.6 (4)
C6A—C7A—C12A—C11A	178.4 (3)	C6B—C7B—C12B—C11B	176.3 (2)
C8A—C7A—C12A—C13A	−178.0 (3)	C8B—C7B—C12B—C13B	−179.2 (2)
C6A—C7A—C12A—C13A	0.3 (4)	C6B—C7B—C12B—C13B	−2.2 (3)
C10A—C11A—C12A—C7A	−0.4 (4)	C10B—C11B—C12B—C7B	1.5 (4)
C10A—C11A—C12A—C13A	177.7 (3)	C10B—C11B—C12B—C13B	−179.9 (3)
C7A—C12A—C13A—O4A	172.8 (4)	C7B—C12B—C13B—O4B	165.4 (2)
C11A—C12A—C13A—O4A	−5.4 (5)	C11B—C12B—C13B—O4B	−13.1 (4)
C7A—C12A—C13A—C14A	−5.6 (4)	C7B—C12B—C13B—C14B	−14.1 (3)
C11A—C12A—C13A—C14A	176.2 (3)	C11B—C12B—C13B—C14B	167.4 (2)
C4A—C5A—C14A—C1A	1.7 (4)	O1B—C1B—C14B—C5B	−175.6 (2)
C6A—C5A—C14A—C1A	−175.0 (2)	C2B—C1B—C14B—C5B	3.5 (3)
C4A—C5A—C14A—C13A	179.1 (3)	O1B—C1B—C14B—C13B	4.0 (3)
C6A—C5A—C14A—C13A	2.4 (4)	C2B—C1B—C14B—C13B	−176.8 (2)
O1A—C1A—C14A—C5A	177.5 (2)	C4B—C5B—C14B—C1B	−0.3 (3)
C2A—C1A—C14A—C5A	−1.8 (4)	C6B—C5B—C14B—C1B	179.2 (2)
O1A—C1A—C14A—C13A	0.1 (4)	C4B—C5B—C14B—C13B	180.0 (2)
C2A—C1A—C14A—C13A	−179.2 (3)	C6B—C5B—C14B—C13B	−0.5 (3)
O4A—C13A—C14A—C5A	−174.2 (4)	O4B—C13B—C14B—C1B	16.2 (4)
C12A—C13A—C14A—C5A	4.1 (4)	C12B—C13B—C14B—C1B	−164.3 (2)
O4A—C13A—C14A—C1A	3.2 (5)	O4B—C13B—C14B—C5B	−164.1 (2)
C12A—C13A—C14A—C1A	−178.4 (2)	C12B—C13B—C14B—C5B	15.4 (3)
C2A—C1A—O1A—C15A	0.7 (4)	C2B—C1B—O1B—C15B	−0.4 (4)
C14A—C1A—O1A—C15A	−178.6 (2)	C14B—C1B—O1B—C15B	178.7 (2)
C16A—C15A—O1A—C1A	177.4 (2)	C16B—C15B—O1B—C1B	−172.6 (2)
C3A—C4A—O2A—C17A	−4.4 (4)	C3B—C4B—O2B—C17B	5.2 (3)
C5A—C4A—O2A—C17A	175.9 (2)	C5B—C4B—O2B—C17B	−173.7 (2)
C18A—C17A—O2A—C4A	179.5 (2)	C18B—C17B—O2B—C4B	175.7 (2)

### Hydrogen-bond geometry (Å, º)

**Table:**

*D*—H···*A*	*D*—H	H···*A*	*D*···*A*	*D*—H···*A*
C8*A*—H8*A*···O3*B*	0.94	2.48	3.234 (3)	137
C8*B*—H8*B*···O3*A*	0.94	2.55	3.304 (4)	137
C11*A*—H11*A*···O4*B*^i^	0.94	2.60	3.325 (3)	135
C11*B*—H11*B*···O4*A*^ii^	0.94	2.46	3.199 (4)	135

Symmetry codes: (i) *x*+1, *y*, *z*; (ii) *x*−1, *y*, *z*.
