# Supplementary figures and images for: Crystal structure of 1,4-dieth­oxy-9,10-anthra­quinone
Source: Acta Crystallogr E Crystallogr Commun. 2015 Jun 24;71(Pt 7):o504–5. doi: 10.1107/S2056989015011901 (PMC4518951; doi:10.1107/S2056989015011901)

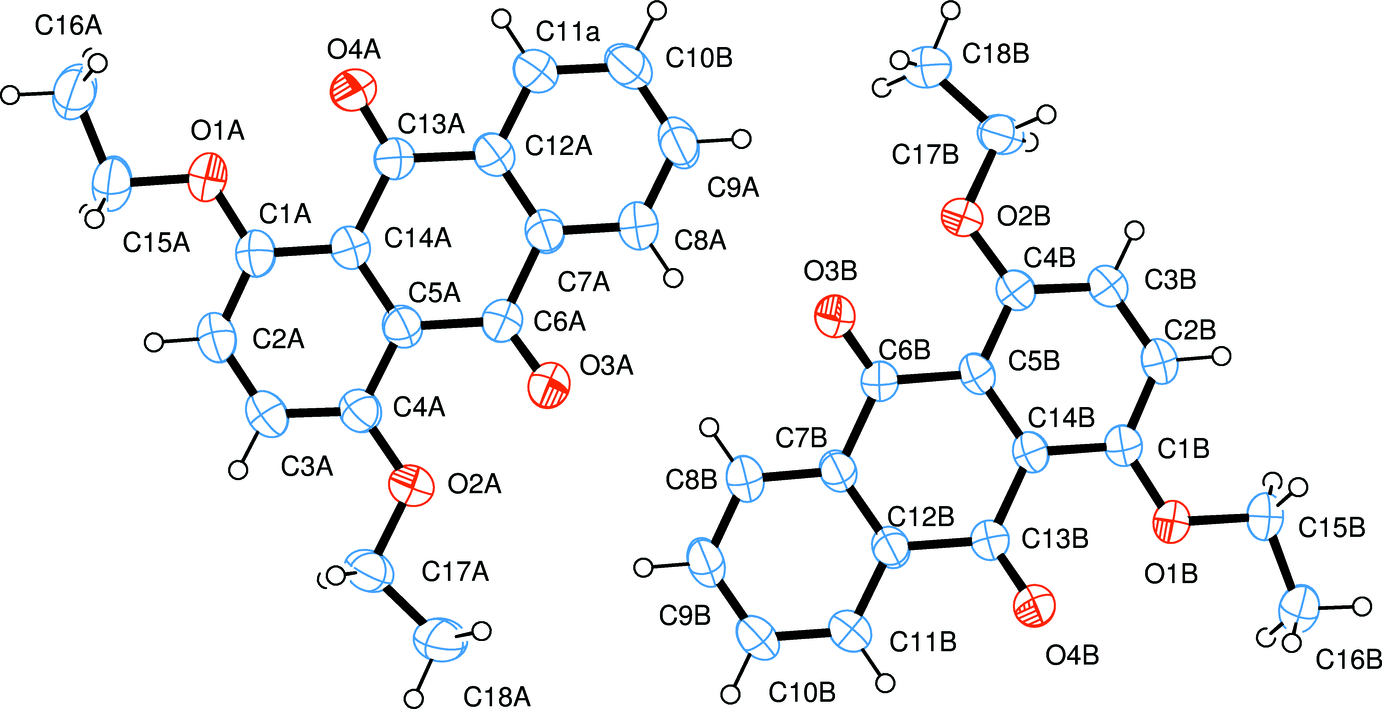

Supplement: Supplementary file 4 [file e-71-0o504-fig1.tif]

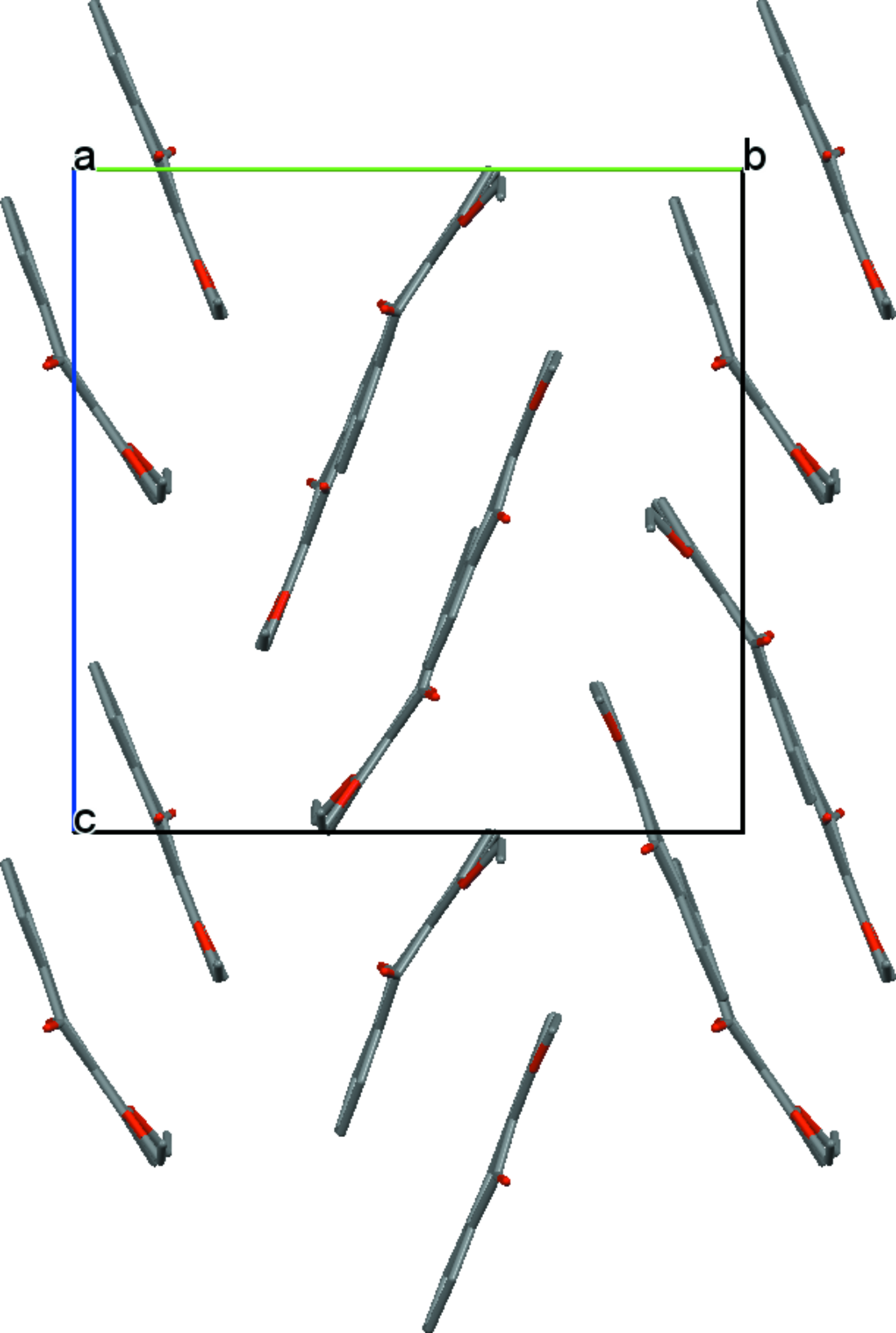

Supplement: Supplementary file 5 [file e-71-0o504-fig2.tif]

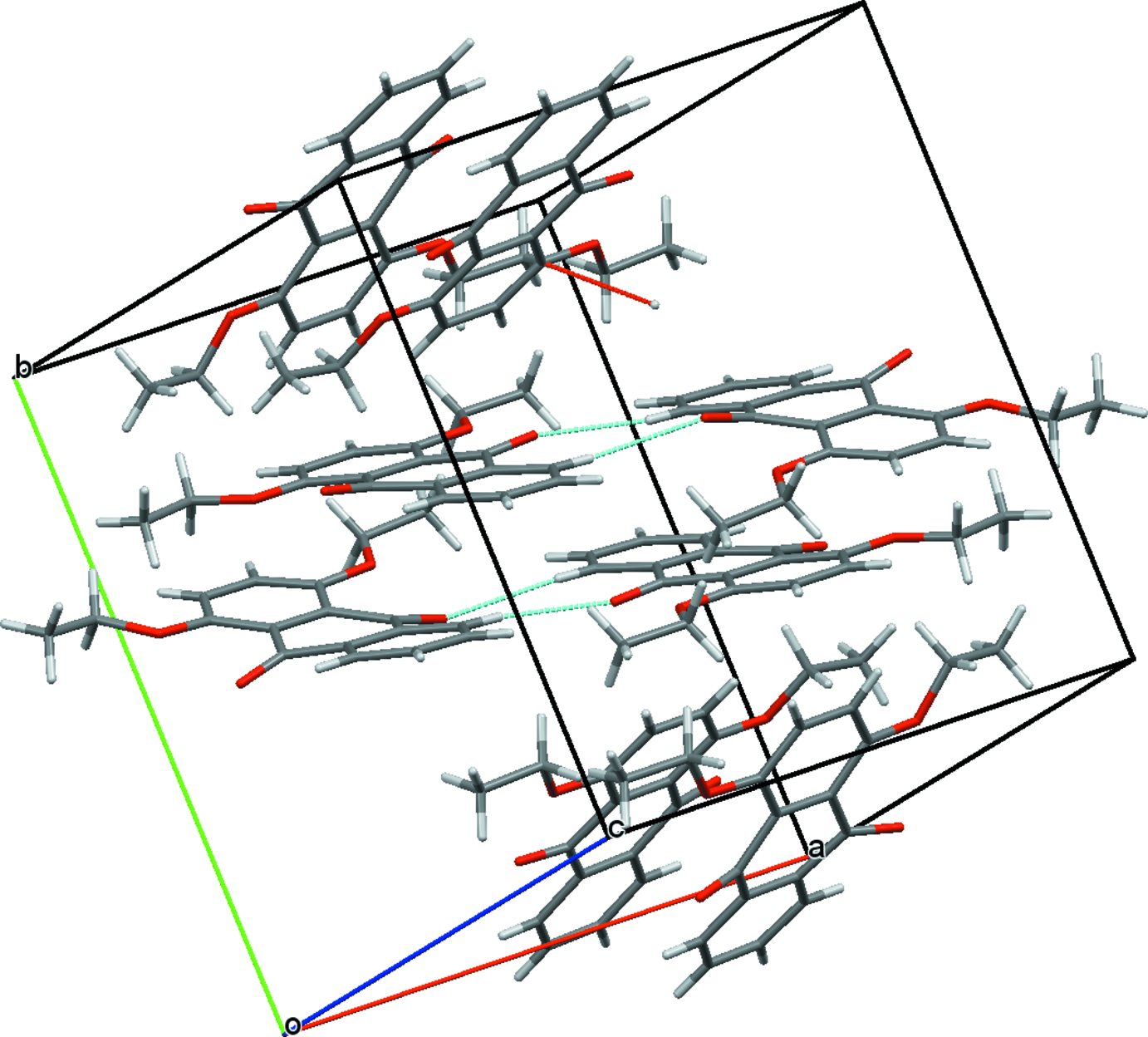

Supplement: Supplementary file 6 [file e-71-0o504-fig3.tif]
